# Effectiveness of the movement control measures during the third wave of COVID-19 in Malaysia

**DOI:** 10.4178/epih.e2021073

**Published:** 2021-09-23

**Authors:** Ahmed Syahmi Syafiq Md Zamri, Sarbhan Singh, Sumarni Mohd Ghazali, Lai Chee Herng, Sarat Chandra Dass, Tahir Aris, Hishamshah Mohd Ibrahim, Balvinder Singh Gill

**Affiliations:** 1 Institute for Medical Research (IMR), National Institutes of Health (NIH), Ministry of Health Malaysia, Setia Alam, Malaysia; 2 School of Mathematical and Computer Sciences, Heriot-Watt University, Putrajaya, Malaysia; 3 Ministry of Health Malaysia, Putrajaya, Malaysia

**Keywords:** COVID-19, Malaysia, Susceptible infected recovered models

## Abstract

**OBJECTIVES:**

Starting in March 2020, movement control measures were instituted across several phases in Malaysia to break the chain of transmission of coronavirus disease 2019 (COVID-19). In this study, we developed a susceptible-exposed-infected-recovered (SEIR) model to examine the effects of the various phases of movement control measures on disease transmissibility and the trend of cases during the third wave of the COVID-19 pandemic in Malaysia.

**METHODS:**

Three SEIR models were developed using the R programming software ODIN interface based on COVID-19 case data from September 1, 2020, to March 29, 2021. The models were validated and subsequently used to provide forecasts of daily cases from October 14, 2020, to March 29, 2021, based on 3 phases of movement control measures.

**RESULTS:**

We found that the reproduction rate (R-value) of COVID-19 decreased by 59.1% from an initial high of 2.2 during the nationwide Recovery Movement Control Order (RMCO) to 0.9 during the Movement Control Order (MCO) and Conditional MCO (CMCO) phases. In addition, the observed cumulative and daily highest numbers of cases were much lower than the forecasted cumulative and daily highest numbers of cases (by 64.4-98.9% and 68.8-99.8%, respectively).

**CONCLUSIONS:**

The movement control measures progressively reduced the R-value during the COVID-19 pandemic. In addition, more stringent movement control measures such as the MCO and CMCO were effective for further lowering the R-value and case numbers during the third wave of the COVID-19 pandemic in Malaysia due to their higher stringency than the nationwide RMCO.

## INTRODUCTION

Since the World Health Organization (WHO) classified the outbreak of coronavirus disease 2019 (COVID-19) as a pandemic on March 11, 2020, there have been more than 170 million confirmed cases and 3.5 million deaths globally as of May 2021 [1]. As of June 1, 2021, there have been 579,462 COVID-19 cases and 2,867 deaths reported in Malaysia [2]. The first wave of the COVID-19 pandemic lasted from January 25 to February 26, 2020, with 22 confirmed cases. Malaysia experienced a subsequent second wave from February 27 to September 19, 2020 [3], followed by a third wave beginning on September 20, 2020 [4].

Several non-pharmaceutical interventions were implemented to control the spread of COVID-19 in Malaysia that included various phases and degrees of movement restrictions such as the Movement Control Order (MCO), Conditional Movement Control Order (CMCO) and Recovery Movement Control Order (RMCO). These measures varied in terms of stringency level; the MCO was the most stringent, followed by the CMCO and RMCO [5,6]. The MCO was implemented on March 18, 2020, and the stringency level was subsequently adjusted based on the progression of COVID-19 cases and situational urgency [5,7]. During the MCO, only essential economic activity was allowed, and no interstate travel and gatherings were permitted. While many economic sectors continued to operate during the CMCO and RMCO with strict standard operating procedures, interstate travel was only allowed during the RMCO phase [5,8]. A study conducted by Gill et al. [9] in 2020 found that the movement control measures were effective at controlling the spread of COVID-19 and flattening the epidemic curve during the second wave of the COVID-19 pandemic in Malaysia. Similar findings have been observed in France, China, and Korea, where movement control measures also successfully controlled the spread of COVID-19 [9-13].

As a result of improvements in the trend of cases toward the end of the second wave of the pandemic in Malaysia, the more restrictive MCO and CMCO were replaced with the less restrictive RMCO. Three weeks following the implementation of the RMCO, Malaysia began to experience a resurgence in daily cases, which marked the beginning of the third wave of the COVID-19 pandemic on September 20, 2020. The WHO had warned that premature lifting or easing of movement restrictions would increase the risk of a resurgence of COVID-19 cases [14-17]. Similar resurgences in the spread of COVID-19 were also observed following the easing of movement restrictions in Germany, Iran, China, and Korea [13].

After an increase in COVID-19 cases during the third wave, the CMCO was re-instituted on October 13, 2020, in the states of Sabah, Selangor, the Federal Territory of Kuala Lumpur, Putrajaya, and Labuan, and it was extended nationwide on November 9, 2020 [18,19]. Gradual increases in the level of stringency of the movement restrictions from the RMCO to MCO during the third wave were instituted to control the spread of COVID-19 (Supplementary Material 1). Hence, it is important to determine the effectiveness of the movement control measures implemented during the third wave of the COVID-19 pandemic in Malaysia to substantiate the need for increasing the degree of stringency [9,20-24].

As was previously determined during the second wave of the pandemic in Malaysia, the effects of movement control measures on the spread of COVID-19 could be assessed by measuring changes in case incidence and the reproduction rate (R-value) [14,25-28]. These indicators are both sensitive and effectively reflect the disease burden and transmissibility of COVID-19. In order to determine changes in the R-value during the outbreak, several studies have reported that compartmental models such as the susceptible-infected-recovered and susceptible-exposed-infectedrecovered (SEIR) models are effective for measuring R-values. Furthermore, these models can accurately forecast the trend of COVID-19 cases. Therefore, they reflect the effects of control measures on the case incidence and transmission dynamics of COVID-19 [9,22]. In this study, we used the R-values generated by an SEIR compartmental model to assess the effects of the various phases of the movement control measures on the number of COVID-19 cases and disease transmissibility during the third wave of the COVID-19 pandemic in Malaysia.

## MATERIALS AND METHODS

### Data source

Daily COVID-19 case data were sourced from the official website of the Ministry of Health (MOH), Malaysia, with a date range of September 1, 2020, to March 29, 2021 [23]. The date range was selected for this study to examine the effectiveness of movement control measures of varying stringency levels during the third wave of the COVID-19 pandemic in Malaysia. Subsequent periods beyond those of this study period were not included in the analysis as the dynamics of disease transmission (after March 29, 2021) varied due to the effects of COVID-19 vaccination and new COVID-19 variants such as the Delta variant [24,25]. In addition, after March 29, 2021, the categorization of movement control measures was changed by the National Recovery Plan, category 1 to category 4, which was not comparable to the classification of the movement control measures during the third wave of the pandemic. Thus, the effectiveness of movement control measures would have been unclear [26]. Due to these reasons, this paper did not examine data from periods beyond March 29, 2021, as doing so would have confounded the effects of the movement control measures during the third wave. In addition, several local and international validated parameters used in the development of the model included the total population of Malaysia and the incubation and infectious period of COVID-19, which were sourced from other studies, while the force of infection and disease transmissibility were calibrated by the model as shown in Table 1.

### Statistical analysis

The SEIR models were developed using the R programming software ODIN interface, which is a modeling interface built and developed by Imperial College London [27-30]. A 7-day moving average of daily cases was used in the development of the model to reduce the variability of the daily number of cases [31-33]. The SEIR model was validated based on the forecast of the fitted model, which was then compared to observed case trends.

The SEIR model was developed based on 3 phases of nationwide movement restrictions during the third COVID-19 wave: (1) the nationwide RMCO (September 1 to October 13, 2020), (2) the nationwide MCO (September 1, 2020 to February 4, 2021), and, finally, (3) the MCO with CMCO (February 5 to March 29, 2021), as shown in Table 2. For the nationwide RMCO phase, the SEIR model was fitted to smoothed daily cases from September 1 to October 13, 2020. Subsequently, the fitted model was used to forecast the trend of cases from October 14, 2020 to March 29, 2021. Next, during the nationwide MCO, the model was fitted from September 1, 2020 to February 4, 2021, (highest daily observed cases) and forecasted the trend of cases from February 5 to March 29, 2021. Finally, the third phase was the MCO and CMCO, for which the model was fitted from February 5 to March 29, 2021.

We estimated and compared the R-values for each phase of movement restrictions. In addition, we determined the forecasted cases for each phase using its respective R-value and compared the forecasted and observed cumulative case rate for each phase as of March 29, 2021. Furthermore, we compared the highest daily forecasted number of cases for each phase with the highest observed number of cases up to March 29, 2021. The R-value and the observed and forecasted cumulative and highest daily number of cases were compared for each phase to determine the effect of the degree of stringency of movement restrictions on outbreak intensity and progression.

### Susceptible-exposed-infectious-removed mathematical model formulation

**Figure 1.**
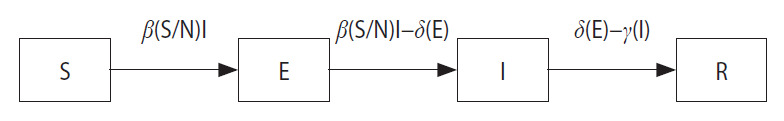
The susceptible-ex posed-infected-recovered (SEIR) model.

The SEIR model developed in this study had 4 state variables as shown in Figure 1—susceptible (*S*), exposed (*E*), infectious (*I*) and removed (*R*)—and 3 recruitment rates: *β* for the force of infection, *δ* for the rate of incubation, and *γ* for the rate of infectiveness. The SEIR model used in this study had the following assumptions: (1) it was a closed population, (2) the entire Malaysian population was initially susceptible, hence *S*_0_=*N*, (3) all individuals were assumed to be equally likely to contract and transmit the virus assuming there was homogenous mixing within the population, and (4) the potential epidemic was likely to be explosive within a short timeframe, and background birth and death rates were therefore not included in the estimation.

Based on the assumptions, the transmission model of COVID-19 in Malaysia was formulated using the SEIR model. Prior to the appearance of symptoms, a COVID-19 patient is mobile and free to interact with other susceptible persons. The force of infection, or the rate at which an infected individual in the population (that has not yet been isolated/quarantined) who comes in contact with susceptible individuals, is governed by beta (*β*). Hence, *β* determines the extent of the transition of individuals from *S* to *E* (*E* represents the proportion of individuals who have been exposed to the virus but are not yet infectious). The duration of an individual remaining in *E* is determined by the length of the incubation period of the virus (1/*δ*), with *δ* denoting the corresponding rate. Exposed individuals (*E*) who subsequently become infectious after the incubation period will then be classified as *I*. Once classified as *I*, the infected individual spreads the virus to other susceptible individuals until he/she is quarantined. The duration of infectiveness (1/*γ*) is determined by its rate (*γ*). Infected individuals who have been diagnosed are subsequently removed (*R*) from the population. The R-value represents the reproduction rate, which is determined by the force of infection over the period of infectiveness (R-value=*β*/*γ*), with *β* representing the force of infection and *γ* indicating the rate of infectiveness [34]. The value of *γ* was determined to be 1/3.95 days, which was obtained from a previous study [29], while the value of *β* was calibrated based on the SEIR model.

### Ethics statement

The study was registered with National Medical Research Register (NMRR-21-597-59440). No ethics approval was required.

## RESULTS

**Figure 2.**
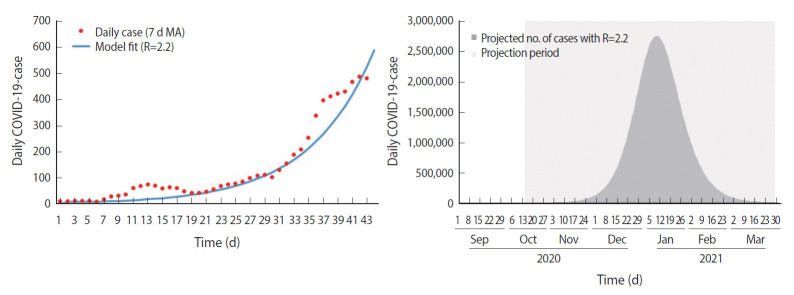
SEIR model fit and forecast for nationwide RMCO phase. SEIR, susceptible-ex posed-infected-recovered; RMCO, recovery movement control order; COVID-19, coronavirus disease 2019; MA, moving average.

The SEIR compartmental model with the best-fit R-value was used to forecast the cumulative and highest daily number of cases during the 3 phases of the movement control measures (nationwide RMCO, nationwide MCO, and MCO with CMCO) and was compared to the observed values. As shown in Figure 2, the estimated R-value was 2.2 during the nationwide RMCO phase, during which the cumulative forecasted number of cases and observed number of cases as of March 29, 2021, were 29,061,753 and 333,545, respectively, showing a 98.9% reduction in cumulative cases. The highest daily forecasted number of cases was observed on January 10, 2020, with 2,762,490 cases compared to the 5,728 cases observed on January 30, 2021, during the RMCO phase. This corresponds to a 99.8% reduction from the peak forecasted number of cases.

**Figure 3.**
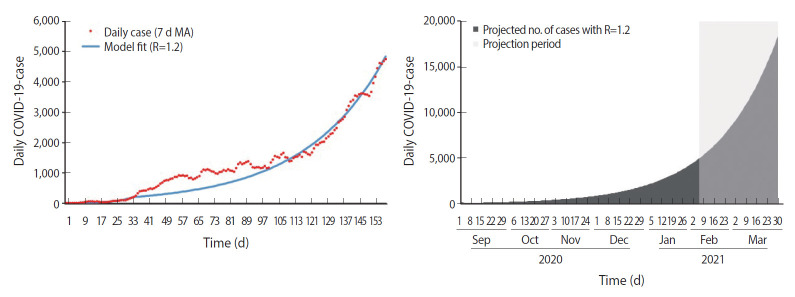
SEIR model fit and forecast for nationwide MCO. SEIR, susceptible-ex posed-infected-recovered; MCO, movement control order; COVID-19, coronavirus disease 2019; MA, moving average.

**Figure 4.**
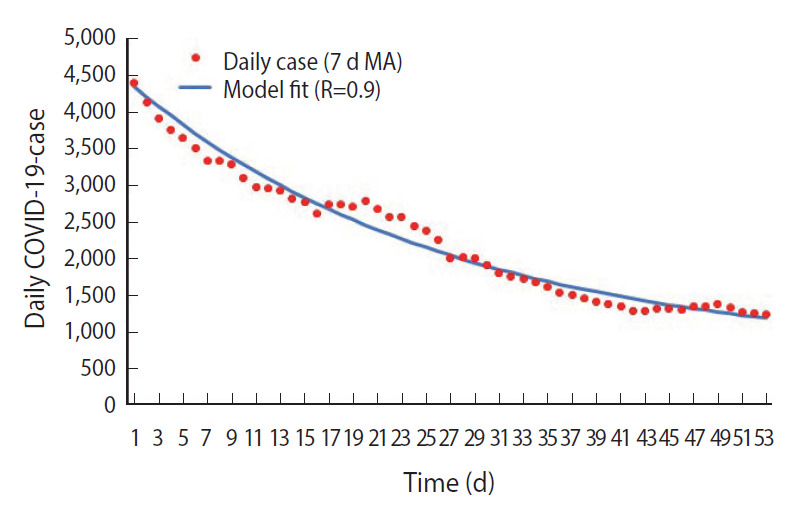
SEIR model fit for MCO with CMCO phase. SEIR, susceptible-ex posed-infected-recovered; MCO, movement control order; CMCO, conditional movement control order; COVID-19, coronavirus disease 2019; MA, moving Average.

As shown in Figure 3, the estimated R-value was 1.2 during the nationwide MCO phase, during which the cumulative forecasted number of cases and observed number of cases as of March 29, 2021, were 937,384 and 333,545, respectively, showing a 64.4% reduction in cumulative cases. In addition, the highest daily forecasted number of cases was observed on March 29, 2021, with 18,331 cases compared to the 5,728 cases observed on January 30, 2021, during the nationwide MCO phase. This corresponds to a 68.8% reduction from the peak forecasted number of cases. Finally, the estimated R-value was 0.9 during the MCO with CMCO phase, as shown in Figure 4.

As detailed above, the R-value decreased from 2.2 to 0.9 during the various phases of movement control restrictions. This shows that the R-value decreased by 59.1% after the introduction of the movement control restrictions. In addition, as shown in Table 2, the decrease in the cumulative number of cases during the third wave ranged from 64.4% to 98.9%. A reduction by 28,728,208 cumulative cases (98.9%) was observed between the forecasted cumulative number of cases with an R-value of 2.2 and the observed cumulative number of cases from October 14, 2020, to March 29, 2021. Similarly, there was a 66.8% to 99.8% reduction in the highest daily forecasted number of cases compared to the observed highest daily number of cases on January 30, 2021.

## DISCUSSION

This study used changes in the R-values generated by an SEIR compartmental model to measure the effects of the various movement control measures in Malaysia on case numbers and disease transmissibility during the third wave of the COVID-19 pandemic. In this study, we found that the movement control measures progressively reduced the number of COVID-19 cases and disease transmissibility during the third wave from September 1, 2020 to March 29, 2021. This was indicated by a 64.4% to 98.9% decrease in the cumulative number of cases during the transition from the nationwide RMCO to MCO and a 59.1% reduction in the R-value from the initial R-value of 2.2 to 0.9, as reported in this study. This can be attributed to the effects of the movement control measures, which essentially reduced the physical contact and mobility of individuals and, in turn, resulted in a decrease in disease transmission [10]. Similar observations were reported across countries in Europe, which showed an average reduction in disease transmissibility of 81% compared to before the MCOs were implemented [35].

During the nationwide RMCO, we found an R-value of 2.2. This could be explained by several factors. First, the RMCO was initially implemented from June 10, 2020, until December 31, 2020, and focused on economic recovery, still allowing interstate travel and permitting certain business sectors to continue to operate [18]. However, following the easing of interstate travel restrictions as well as elections in Malaysia, the daily number of COVID-19 cases started to increase as reflected by the estimated R-value of 2.2 in this study [36,37]. Similarly, studies conducted in Germany, Spain, Japan, and China found that premature easing of movement control measures led to increases in the mobility of the population and physical interaction which in turn increased the risk of disease transmission, therefore resulting in a resurgence of COVID-19 cases [13,20,38-42]. In addition, a previous study found that mobility following the easing of interstate travel restrictions resulted in a wider spread of disease into unaffected areas, leading to massive outbreaks [12].

The spread of COVID-19 continued to increase due to the R-value being above 1, indicating that it was self-sustaining. Therefore, in order to reduce the R-value to control the outbreak, the nationwide MCO was implemented in January 2021 [13]. This resulted in a 45.5% reduction in the R-value from 2.2 (nationwide RMCO) to 1.2 (nationwide MCO) as reported in this study. These findings can be attributed to the nationwide MCO’s higher degree of stringency compared to the RMCO, since intrastate and interstate movement restrictions were expanded during the MCO phase [5,12,42,43].

The nationwide MCO was implemented for 2 months beginning in January 2021 and successfully decreased the trend of COVID-19 cases [5,44]. In response to the decreasing trend of cases and the need to strike a balance between lives and livelihood, the government implemented the CMCO in March 2021 with the aim of minimally disrupting socioeconomic sectors to promote economic recovery alongside the continuation of outbreak control measures [20,45,46]. Our study found that the transition from the nationwide MCO to the MCO with targeted CMCO further reduced the R-value from 1.2 to 0.9. Although the nationwide MCO was stricter than the CMCO, the R-value decreased to a lower level than during the nationwide MCO. This could be attributed to the initial effect of nationwide MCO on reducing the transmissibility of COVID-19 since the outbreak had reached its peak during the nationwide MCO, and the trend of cases subsequently began to decrease. As a result, the implementation of targeted movement restriction measures such as the CMCO following the nationwide MCO was sufficient for controlling the outbreak as there were fewer localized clusters to target. This is supported by evidence from other studies showing that targeted movement control measures were effective for controlling the spread of COVID-19 when the trend of cases was decreasing [5,20,46-48].

In this study, we also found that stringent movement control measures had a substantial impact on decreasing the R-value during the pandemic. The MCO and CMCO resulted in an overall decrease in the R-value by 59.1% (from 2.2 to 0.9). As a result of the decrease in the R-value due to more stringent movement control measures, the observed cumulative and daily highest numbers of cases were much lower compared to the forecasted cumulative and daily highest numbers of cases, by 64.4-98.9% and 68.8-99.8%, respectively, during the third wave of the COVID-19 pandemic in Malaysia. These findings show that the timely implementation of stringent movement control measures prevented the healthcare system from becoming overwhelmed [13]. Delays in instituting more stringent measures for controlling the spread of COVID-19 could have resulted in uncontrolled outbreaks that in turn may have overburdened the healthcare system as observed in other countries [49]. Similarly, a study involving 16 countries that conducted mathematical modeling also found that stringent movement control measures reduced R-values to below 1, which in turn decreased the trend of cases, hospitalizations, and deaths [46].

In addition, this study showed that the adjustments made to the various phases of the movement control measures were justified and corresponded to the considerations for implementing and adjusting public health and social measures (PHSM) in the context of COVID-19—an interim guideline by the WHO [50]. This guideline suggests that adjustments to PHSM such as movement control measures should be based on community transmission (level 1 to 4, which indicates low to very high incidence rates of COVID-19 infections) and health response capacity (level 1 to 3, which indicates adequate to limited response capacity) to provide an overall assessment of the situational level which ranges from level 0 (no community transmission with adequate response capacity) to level 4 (uncontrolled epidemic with limited response capacity). This study found that the implementation of stringent movement control measures such as the nationwide MCO and CMCO during the third wave of the COVID-19 pandemic in Malaysia corresponded to high levels of community transmission and limited public health response capacity. More specifically, the nationwide RMCO, nationwide MCO, and MCO with CMCO corresponded to situational level 1, levels 2 to 4, and level 3, respectively. Furthermore, since decisions concerning implementing and adjusting movement control measures in various phases were guided by the WHO interim guidelines, which were based on several indicators, the movement control measures taken in Malaysia effectively struck a balance between lives and livelihood.

This study was the first to assess the effectiveness of different phases of movement control measures during the third wave of the COVID-19 pandemic in Malaysia using an SEIR model. It was also the first to estimate the decrease in the cumulative number of cases and highest daily number of cases resulting from the implementation of movement control measures. The strengths of this study include the use of smoothed data for the development of the SEIR model. The use of smoothed data reduced noise, therefore making the model more accurate and sensitive. In addition, the model was parameterized with validated local and international parameters.

In conclusion, this study found that stringent movement control measures such as the MCO and CMCO were more effective at reducing the R-value and the number of cases than the nationwide RMCO during the third wave of the COVID-19 pandemic in Malaysia. Therefore, adjustments to movement control measures must be made with caution to prevent resurgence of COVID-19 cases.

## Figures and Tables

**Table 1.** Corresponding parameters and their respective values in the model

Parameter	Description	Value	Source
n	Total population of Malaysia	32,000,000 population	[27]
1/*δ*	Incubation period of COVID-19	5.20 d	[28]
$\frac{1}{\gamma }$	Infectious period of COVID-19	3.95 d	[29]
*β*	Force of infection		Calibrated
R-value	Transmissibility		Calibrated

COVID-19, coronavirus disease 2019.

**Table 2.** Estimated R-values generated by the SEIR model for three scenarios

Scenario	Nationwide RMCO	Nationwide MCO	MCO with CMCO^1^
Model fit period	Sep 1, 2020 to Oct 13, 2020	Sep 1, 2020 to Feb 4, 2021	Feb 5, 2021 to Mar 29, 2021
Model forecast period	Oct 14, 2020 to Mar 29, 2021	Feb 5, 2021 to Mar 29, 2021	-
R-value	2.2	1.2	0.9
Cumulative forecasted no. of cases as of Mar 29, 2021 (a)	29,061,753	937,384	-
Cumulative observed no. of cases as of Mar 29, 2021 (b)	333,545	333,545	333,545
Difference between forecasted and observed cumulative no. of cases as of Mar 29, 2021 (a-b) (%)	28,728,208 (98.9)	603,839 (64.4)	-
Highest daily forecasted no. of cases as of Mar 29, 2021 (c)	2,762,490	18,331	-
Highest daily observed no. of cases as of Mar 29, 2021(d)	5,728	5,728	-
Difference between highest daily no. of cases and forecasted and observed no. cases as of Mar 29, 2021 (c-d) (%)	2,756,762 (99.8)	12,603 (68.8)	-
Reduction in R-value from RMCO to MCO	45.5		
Reduction in R-value from MCO to MCO with CMCO		25.0	
Overall reduction in R-value	59.1		

SEIR, susceptible-exposed-infected-recovered; RMCO, recovery movement control order; MCO, movement control order; CMCO, conditional movement control order.

1 This phase only involved estimating the R-value during the model fit period.
