# Supplementary material for: Effectiveness of the movement control measures during the third wave of COVID-19 in Malaysia
Source: Epidemiol Health. 2021 Sep 23;43:e2021073. doi: 10.4178/epih.e2021073 (PMC8891114; doi:10.4178/epih.e2021073)
Supplement: Supplementary file 1 [file epih-43-e2021073-suppl.docx]

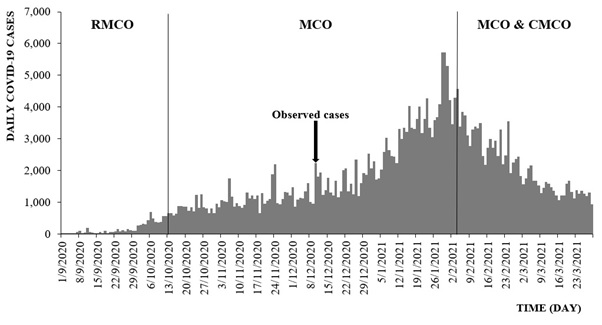


**Supplementary Material 1.** Daily COVID-19 cases across various movement control order, September 1, 2020 to March 29, 2021, Malaysia. RMCO, recovery movement control order; MCO, movement control order; CMCO, conditional movement control order; COVID-19, coronavirus disease 2019.
